# Factors influencing paramedics’ and emergency medical technicians’ level of knowledge about the 2015 basic life support guidelines

**DOI:** 10.1186/s12873-021-00474-8

**Published:** 2021-07-12

**Authors:** Celal Levent Kayadelen, Ayşe Nilgün Kayadelen, Polat Durukan

**Affiliations:** 1 Emergency Department, Kahramanmaras Elbistan State Hospital, Karaelbistan Neighbourhood, Istiklal Street, 9, Elbistan, KMaras Turkey; 2 Adana, Turkey; 3 Medicalpark Gaziantep Hospital, Gaziantep, Turkey

**Keywords:** Basic life support applications, Emergency medical technician, Cardiopulmonary resuscitation, Paramedic

## Abstract

**Background:**

This study aimed to determine the cardiopulmonary resuscitation (CPR) knowledge level and factors affecting the current CPR knowledge level among EMTs and paramedics working in the Kayseri 112 emergency health system.

**Methods:**

This survey aimed to reach all paramedics and emergency medical technicians working in the 112 emergency health system in Kayseri province. The data collection consists of sociodemographic/occupational characteristics, CPR training and practice characteristics and 20 four-choice CPR knowledge questions. Multiple linear regression analysis was used to determine the independent variables that affect the number of correct answers given to the questionnaire.

**Results:**

305 healthcare professionals were included in this study. 57.0% (*n* = 174) of the participants were female and 56.1% were under the age of 30. It was found that 65.6% (*n* = 200) of the healthcare professionals were EMTs, and 48.6% (*n* = 148) had been working for 6–10 years. The mean number of correct responses based on the 20 questions asked was 12.76 ± 3.11. In multivariable analysis, it was determined that having received training on CPR after 2015, having participated in a course or seminar on CPR in the last 3 months and having practiced defibrillation/cardioversion during CPR significantly increased the level of knowledge regarding CPR (respectively, *p* < 0.01, *p* = 0.025, *p* = 0.045).

**Conclusion:**

CPR trainings, which have been received recently and based on the current guidelines, increase the level of CPR knowledge and the increasing knowledge level affects the use of defibrillation/cardioversion. Improving training increases knowledge and practice.

## Background

Cardiopulmonary resuscitation (CPR) is a set of emergency interventions aimed to re-start, with external interference, the respiration and circulation in cases of cardiopulmonary arrest and to reinstate respiratory and circulatory functions that have been disrupted. CPR is simple, but requires training and experienced person or persons [1, 2]. Proper intervention in cardiac arrest by healthcare professionals with Basic Life Support (BLS) has been shown to have significant impacts in saving lives [3]. Up-to-date knowledge of the practitioner of CPR and effective and adequate CPR are directly related to the survival of the patient [3, 4].

Studies show that healthcare professionals prove to be insufficient in compliance with the recommendations of current CPR guidelines and their CPR applications are ineffective [5–11]. The insufficient level of survival after the application of CPR shows that the skill level of BLS practitioners has to be improved and the improved level has to be sustained [12]. As emergency medical technicians (EMT) and paramedics working in the 112 emergency aid system are the people most likely to encounter a cardiopulmonary arrest patient outside the hospital, they are the person whose skills and knowledge should have the highest level of compliance with the current CPR guidelines. While there are studies in the literature with regard to the level of knowledge on CPR among different healthcare professionals, studies that focus specifically on EMTs and paramedics who are primarily assigned for pre-hospital CPR are quite limited.

Assessment of healthcare professionals' level of knowledge regarding CPR, their preferences during the implementation of CPR, their level of compliance with the current guidelines and internationally-recommended algorithms and the factors affecting it serve as a guide to achievement of the most successful treatment outcomes. This study aimed to determine the CPR knowledge level and factors affecting the current level of knowledge regarding CPR among EMTs and paramedics working in the Kayseri 112 emergency health system.

## Methods

The population of this survey research consists of EMTs and paramedics working in Kayseri Command Control Center and 112 Emergency Healthcare Stations from October 1 to December 31, 2017. This study is approved by Clinical Research Ethics Committee of Erciyes University, Kayseri, Turkey No. 2017/500, September 15, 2017 (IRB No. 2017/500). Verbal consent was obtained from all participants.

### Participants

EMTs and paramedics working in Kayseri Command Control Center and 112 Emergency Healthcare Stations in relevant dates were included in the study.

Eligibility criteria

- On duty at the time of the study,
- Working in EMT or parametric staff,
- Working in Kayseri Command Control Center and 112 Emergency Healthcare Stations,
- Approving to participate in the study

Participants who did not meet the inclusion criteria were excluded from the study. Since it was difficult to do the survey with all paramedics and EMTs in Kayseri, we preferred to use a convenience sample rather than a sample size calculation, depending on our availability.

### Data collection

The participants filled in the data forms on their own, under surveillance. During the data collection, the participants were taken to a quiet environment as much as possible. A maximum of 25 min was given to fill in the questionnaire, which consisted of 30 questions in total. During the survey, answering questions with other participants or obtaining information from other sources was not allowed.

### Data collection tool and measurements

The data collection form used in the study consists of two parts. The first part consists of sociodemographic and occupational characteristics (age, gender, education status, total working time, the number of CPRs applied in the last 6 months, whether s/he applied defibrillation/cardioversion during CPR or not, training history on CPR and how s/he updates his/her knowledge on CPR), while the second part consists of 20 four-choice knowledge questions where only one option is correct, the preparation of which is based on the AHA's CPR and Emergency Cardiovascular Care Guidelines 2015 and the use of basic life support knowledge (Annex 1).

In the study, the number of correct responses given to knowledge questions was the dependent variable, while all other parameters were considered as independent variables.

### Primary outcome of the study

Number of correct responses given by EMT and paramedics to questions about CPR.

### Secondary outcomes of the study

Features of CPR practice and parameters affecting the level of CPR knowledge.

### Emergency medical system in Kayseri (Turkey)

Emergency health services in Turkey are managed by the “Emergency Health Services General Directorate” under the Ministry of Health. In any emergency health situation, people can dial the 112 toll-free phone number 24 h a day and reach the command control center in that province. In such a case, a fully equipped ambulance with an emergency physician, a paramedic, and an emergency medical technician is assigned from the nearest station to the scene, and it is provided to reach the scene of the incident. Thus, the necessary medical intervention is carried out with the patient or the injured at the scene and the patient is then transferred to the hospital if necessary. In addition to patient intervention, EMTs and paramedics also have responsibilities such as control of the scene, keeping records, maintenance and use of emergency vehicles, materials, tools [13, 14].

### Trainings received by paramedics and EMTs

In-service training required for paramedics and EMTs in Turkey are as follows: Basic module training (40 h), trauma resuscitation course (32 h), adult advanced life support course (20 h), advanced life support course in children (28 h), ambulance driving techniques training (32 h), neonatal resuscitation program (72 h), emergency health services training program (96 h), UMKE (national medical rescue team) basic training (60 h).

Within these trainings, emergency case management, cardiac arrest management, arrhythmias, peri-arrest arrhythmias, emergency chest pain and acute coronary syndrome, advanced life support in special cases (anaphylaxis, asthma, drowning in water, stroke (cerebrovascular event), hypo-hyperglycemia, heat and cold-related emergencies, electric shock, lightning strike, cardiac arrest during pregnancy, approach to intoxication) are explained in detail and with practical applications [13, 14].

### Statistical analysis

In this study, SPSS 23.0 (IBM) was used in the analysis of the data. Central and prevalence criteria such as number, percentage, smallest and largest values, standard deviation, average and median were used in the creation of descriptive statistics. Compatibility of the numerical variables with the normal distribution was tested visually (histogram) and analytically (Shapiro–Wilk) and, to determine the discrepancy between the independent variables, ANOVA (post-hoc LSD test) was used on variables compatible with the normal distribution theory, while Kruskal–Wallis and Mann–Whitney U tests were used on variables incompatible with the normal distribution theory. The value of *p* below 0.05 was considered significant. Multiple regression analysis was conducted to determine the factors affecting the level of knowledge. For this purpose, we developed an explanatory/predictive multiple linear regression model. We included in the model the independent variables showing a univariate relationship with a *p* value < 0.1. A backward selection was performed.

## Results

The number of paramedics and EMTs working at Kayseri Command Control Center and 112 Emergency Healthcare Stations on the specified dates was 386. The 305 paramedics and EMTs we could reach completed the questionnaire. (Response rate: 79%). 57.0% (*n* = 174) of the participants were female and 56.1% were under the age of 30. It was found that 65.6% (*n* = 200) of the healthcare professionals were EMTs, and 48.6% (*n* = 148) had been working for 6–10 years. While 2.3% of the participants had never attended a course/seminar, the date of the most recent course/seminar attended by 22.6% was more than 12 months ago.

The mean number of correct responses based on the 20 questions asked was 12.76 ± 3.11. A statistically significant difference was recorded between CPR training and the time of latest course/seminar participation and number of correct responses (*p* < 0.05). In the study, the number of correct responses of those who received CPR training in 2015 and after are significantly higher than those who did before 2015. In addition, the level of knowledge among those who participated in a course/seminar on CPR in the last 3 months was significantly higher than those who did in the last 6 and 12 months. No statistically significant difference was recorded between sex, age, profession, duty period, time spent in the unit, the number of CPR applications, status regarding the application of defibrillation/cardioversion during CPR and the latest CPR guideline reading time and number of correct responses (*p* > 0.05) (Table 1).

**Table 1:** Univariate analysis of the number of correct responses with regard to characteristics of study population

Variables	n (%)	Correct response	*p*
*Sex*			
Female	174 (57.0%)	12.8 ± 3.5	0.81*
Male	131 (43.0%)	12.7 ± 2.8	
*Age*			
< 25	91 (29.9%)	13.0 ± 2.9	0.53***
25–30	80 (26.2%)	12.9 ± 3.3	
> 30	134 (43.9%)	12.5 ± 3.1	
*Profession*			
EMT	200 (65.6%)	12.5 ± 3.1	0.07*
Paramedic	105 (34.4%)	13.2 ± 3.1	
*Duty period*			
≤ 5 years	84 (27.5%)	12.8 ± 2.9	0.46***
6–10 years	148 (48.6%)	12.6 ± 3.3	
≥ 11 years	73 (23.9%)	13.1 ± 2.9	
Time spent in the unit			
≤ 5 years	175 (57.4%)	12.7 ± 3.2	0.44****
6–10 years	102 (33.4%)	12.8 ± 3.2	
≥ 11 years	28 (9.2%)	13.5 ± 2.6	
*CPR training period*			
Before 2015	222 (72.8%)	12.4 ± 3.1	< 0.01*
2015 and later	83 (27.2%)	13.8 ± 2.9	
*Number of CPR applications in the last 6 months*			
≤ 10	281 (92.2%)	12.8 ± 3.1	0.82**
11 and above	24 (7.8%)	12.6 ± 3.7	
*Defibrillation / cardioversion during CPR*			
Practicing	177 (58.0%)	13.0 ± 3.0	0.09*
Non-practicing	128 (42.0%)	12.4 ± 3.3	
*Most recent participation in course/seminar*			
In the last 3 months	36 (11.8%)	13.9 ± 3.0^a^	0.02***
In the last 4–6 months	78 (25.6%)	12.8 ± 3.1^a^	
In the last 7–12 months	86 (28.2%)	12.6 ± 3.2^a^	
*Most recent CPR guide reading*			
Never read	36 (11.8%)	12.8 ± 3.2	0.10***
Read in the last 6 months	77 (25.2%)	13.5 ± 3.0	
Read in the last 7–12 months	149 (49.9%)	12.5 ± 3.1	
Read more than 12 months ago	43 (14.1%)	12.23 ± 3.0	

*CPR* cardiopulmonary resuscitation, *EMT* emergency medical technician, *SD* standard deviation

Number of correct responses are given as mean ± standard deviation

*Independent samples t test, **Mann Whitney U test, ***one-way ANOVA, ****Kruskal–Wallis test

^a^A Post-Hoc LSD test was performed to determine which group the difference originated from. There is a statistically significant difference between the groups indicated by different letters (*p* < 0.05)

Multivariable analysis of variables affecting the participants' level of knowledge regarding CPR is given on Table 2. The model detected a statistically significant relationship between the level of knowledge regarding CPR and the status of defibrillation/cardioversion application during CPR, CPR training and most recent participation in a course/seminar. Accordingly, the number of correct responses of participants who have implemented defibrillation/cardioversion during CPR is approximately 0.4 points higher than the overall average, and 0.6 points higher among those who received CPR training in and after 2015. In addition, the number of correct responses of those who participated in courses/seminars in the last 3 months was approximately 1 point higher than those who did in the last 12 months. Other variables included in the multiple regression model: profession (*p* = 0.229), and most recent CPR guide reading (*p* = 0.466) were found as non-significant. The validity of the model was checked by verifying the linearity, the normality of the residuals and the homoscedasticity.

**Table 2:** Multivariable analysis of the number of correct responses with regard to characteristics of study population (Backward stepwise, step 3)

	b	S(b)	T	*p*	CI for b	
					Lower limit	Upper limit
Fixed	12.86	.26	49.92	< 0.01	12.35	13.37
*Defibrillation/cardioversion during CPR (ref: No application)*						
Presence of application	.41	.20	2.02	0.045	.01	.81
*CPR training period (ref: before 2015)*						
After 2015	.58	.22	2.69	< 0.01	.16	1.01
*Most recent participation in course/seminar (ref: within the last 12 months)*						
In the last 3 months	1.03	.46	2.26	0.025	.13	1.92

B, Regression Coefficient; S(b), Standard error of regression coefficient; *CI* Confidence Interval; R^2^, .08; *CPR* cardiopulmonary resuscitation

## Discussion

Some updates are made on CPR by periodically-published international guidelines. The regular repetition of these guidelines could increase the competence of healthcare providers on current practices, and training programs organized in this regard could be useful in improving the frequency of success achieved in CPR applications. This study examined the level of knowledge among EMTs and paramedics working in Kayseri 112 emergency health system regarding CPR. It was determined that having received training on CPR after 2015, having participated in a course or seminar on CPR in the last 3 months and having practiced defibrillation/cardioversion during CPR significantly increased the level of knowledge regarding CPR.

Several other studies also reported that training in the last 3 or 6 months significantly increased the level of CPR knowledge [15–19]. In a systematic review where 34 different studies were evaluated, it was determined that, regardless of the time of training, people who had received CPR training at some point had significantly higher levels of CPR skills compared to those who had never received it [20]. Providing healthcare professionals up-to-date courses/seminars on CPR at any time, especially within 3 months, should be among the interventions that aim to increase their level of knowledge and skills.

The questions addressed to the people included in this study were taken from the currently published 2015 AHA guidelines and it was determined that the level of CPR knowledge was significantly higher among those who had received CPR training after 2015. In a similar study, Kirazaldı determined that the participants most frequently answered questions in the 2015 AHA guidelines incorrectly, while healthcare professionals who follow the current CPR guidelines had a higher frequency of correct answers for questions on CPR [21]. In his study with EMTs, Brown reported that knowledge of the current guidelines had an impact on more accurate and effective implementation of CPR [22]. In accordance with our study, different studies also report that informative courses/seminars following the publication of the current guidelines have a significant positive impact on healthcare professionals' level of knowledge [18, 23]. In our study, various factors such as the considerably low frequency of following the current guidelines among the participants, recent participation in training, courses and seminars being much longer than the recommended interval of 6 months may have had an impact on the high frequency of incorrect answers. In addition, the fact that questions evaluated current knowledge. In addition, the fact that information questions evaluated in our study examined information featured in the AHA guideline published in 2015 may have resulted in people who had received training after 2015 answering questions on current knowledge more accurately. By supporting this conclusion, Veronese et al. reported that emergency services professionals attending the seminar before the data of publication of the current guidelines had an adverse impact on the level of knowledge [24].

Another important finding of our study was that people who have a better average in the questionnaire provided defibrillation more often than others. In previous studies in accordance with our study, the authors reported that people who had recently received recurring training on CPR had a higher level of CPR knowledge and performance and that this indirectly increased the frequency of defibrillation/cardioversion device use [25, 26]. In another study it was reported that the level of knowledge on CPR had a significant impact on CPR skills [24]. Furthermore, it was shown in previous studies that CPR skills are atrophied in shorter time than the level of knowledge [27, 28]. In order to implement defibrillation/cardioversion, it is necessary to know both the operating principle of the device and the cases in which the procedure should be performed. As people with a good level of CPR knowledge are more in command of these characteristics, they may have implemented these procedures at times required.

We wanted to compare the CPR knowledge level found in our study with studies conducted in other regions. However, the fact that the questions and criteria used in evaluating the level of knowledge among the studies were completely different and the research groups were composed of different occupational groups prevented this effort.

There are limited aspects of this study. During the collection of data, recall bias may have occurred on the part of the participants and people may have misremembered some dates and experiences. There may have been contamination between the people who participated in the study and those who had not yet done so during the data collection process. As the questions on CPR were not taken from a standard scale that was previously subjected to validity and reliability study, their competence in determining the level of CPR knowledge is contentious. In addition, the curriculum quality of the schools from which the participants graduated and the quality of the education on CPR were not evaluated. CPR knowledge can be more permanent after trainings in which participants can actively take part, visualization is highlighted and informative videos and evidence-based resources are used [21, 29, 30]. These cases may have been among the factors affecting the level of knowledge and caused some misinterpretations in the results of our study. Although our study shows, in accordance with the literature, the boosting impact of a recent information update on the level of knowledge, an optimal training interval could not be obtained by examining the information of those who regularly receive training at the same intervals. In this regard, the most suitable training intervals for the memorization of information and skills can be determined with studies including groups of people who had received the same training at different periods and where intergroup comparisons can be conducted.

## Conclusion

In conclusion, this study determined that the training received after the current guideline is published and especially in the last 3 months had a positive impact on the EMTs and paramedics' CPR knowledge and their frequency of using defibrillation/cardioversion. Planning of training programs aiming to update and develop the knowledge and skills of healthcare professionals working in emergency interventions at the intervals of 3 months and informing the people of innovations by following the current guidelines may help improve their level of correct information on CPR and their competence in practices with vital importance such as defibrillation during CPR.

## Data Availability

The data used during the current study are available from the corresponding author on reasonable request.

## Abbreviations

AHA: The American Heart Association
ANOVA: Analysis of variance
BLS: Basic life support
CPR: Cardiopulmonary resuscitation
EMT: Emergency medical technicians
ERC: European Resuscitation Council
ILCOR: The International Liaison Committee on Resuscitation
